# Foliar Accumulation of Melatonin Applied to the Roots of Maize (*Zea mays*) Seedlings

**DOI:** 10.3390/biom9010026

**Published:** 2019-01-12

**Authors:** Young Ha Yoon, Minjae Kim, Woong June Park

**Affiliations:** Department of Molecular Biology, Institute of Nanosensor & Biotech, Dankook University, Cheonan-si 31116, Korea; kakaru0122@naver.com (Y.H.Y.); minjae9381@nate.com (M.K.)

**Keywords:** melatonin, foliar accumulation, abscisic acid, salicylic acid, transpiration, maize, *Zea mays*

## Abstract

Plants absorb melatonin from the environments as well as they synthesize the regulatory molecule. We applied melatonin to the roots of maize (*Zea mays*) seedlings and examined its accumulation in the leaves. Melatonin accumulation in the leaves was proportional to the exogenously applied concentrations up to 5 mM, without saturation. Time-course analysis of the accumulated melatonin content did not show an adaptable (or desensitizable) uptake system over a 24-h period. Melatonin accumulation in the leaves was reduced significantly by the plant hormones abscisic acid (ABA) and salicylic acid (SA), which commonly cause stomatal closure. The application of ABA and benzo-18-crown-6 (18-CR, a stomata-closing agent) induced stomatal closure and simultaneously decreased melatonin content in the leaves. When plants were shielded from airflow in the growth chamber, melatonin accumulation in the leaves decreased, indicating the influence of reduced transpiration. We conclude that melatonin applied exogenously to the root system is absorbed, mobilized upward according to the transpirational flow, and finally accumulated in the leaves.

## 1. Introduction

Melatonin (*N*-acetyl-5-methoxytryptamine) was first identified in plants in 1995 [1,2]. Since then, melatonin has been detected in diverse plants. The established and hypothesized physiological roles of melatonin in plants have been well documented in some reviews [3,4]. Melatonin is recognized as a ubiquitous molecule with physiological roles in defense responses against biotic and abiotic stresses, and in the regulation of plant growth and development [5,6,7,8]. Melatonin mediates plant innate immunity [9] and alters gene expression involved in defense responses induced by both pathogen-associated molecular patterns and effectors [10]. Melatonin treatment improves tolerance to cold stress [11] and water stress [12], delays senescence induced by the drought stress [13], and protects plants from oxidative stress [14]. Furthermore, melatonin interacts with plant hormones [8], e.g., auxin [15], gibberellic acid (GA), cytokinin and abscisic acid (ABA) [16,17], in diverse biological processes. For a long time, it was unclear how plants sense melatonin. A recent study in *Arabidopsis thaliana* has shown that melatonin-induced stomatal closure is mediated by a G protein-coupled receptor, called CAND2/PMPTR1 [18]. Melatonin shows specific binding to CAND2/PMPTR1 with a dissociation constant (*K*_d_) value of 0.73 ± 0.10 nmol/L; the *cand2/pmptr1* mutant was impaired in melatonin response, implicating that CAND2/PMPTR1 is a melatonin receptor. The identification of a specific melatonin receptor in plants has further enhanced the identity of melatonin as a key regulatory substance [19].

Plants synthesize melatonin de novo from tryptophan in four steps [20]. Tryptophan is decarboxylated and converted to serotonin by hydroxylation, and then further converted to melatonin by acetylation and methylation. However, the order of these reactions and the involved enzymes vary [20,21]. Thus, more than four types of genes are involved in melatonin biosynthesis, indicating multiple pathways or a biosynthetic network [20,21].

In addition to de novo biosynthesis in plants, melatonin is also absorbed from the environment. The application of ^3^H-labeled melatonin to red goosefoot (*Chenopodium rubrum*) plants showed that melatonin is absorbed and maintained at a stable level for 37 h [22]. In another study, the application of melatonin (5 μM) to the growth medium of water hyacinth (*Eichhornia crassipes*) for five days showed melatonin accumulation in leaves, based on high-performance liquid chromatography analysis of leaf extracts [23]. Similarly, imbibition of lupin (*Lupinus albus*) cotyledons at various concentrations of melatonin (1 nM–1 μM) revealed that melatonin accumulation was proportional to the concentration of melatonin applied [24]. Barley (*Hordeum vulgare*) leaves also showed a concentration-dependent uptake of melatonin [25]. In the concentration range used in the uptake assay (0.1 μM–1 mM), melatonin accumulation did not reach saturation [25]. Several additional reports of exogenously applied melatonin [11,26,27,28,29,30,31] suggest that plants absorb melatonin from environment. A recent study shows the distribution of absorbed melatonin in St. John’s wort (*Hypericum perforatum*) using images obtained by a novel technique, quantum dot nanoparticles, showing the absorption and movement of exogenously applied melatonin [32].

A previous report on water hyacinth demonstrated that melatonin was absorbed by the roots and transported to the leaves [23]. However, detailed information on melatonin movement in plants is limited. In this work, we examined the characteristics of foliar accumulation of melatonin in maize (*Zea mays*) seedlings, when melatonin was applied exogenously to the roots. We also tested the influence of several plant hormones, other chemicals, and conditions affecting water flow driven by transpiration. Based on the results, we discuss the effect of transpiration on the absorption and foliar accumulation of exogenously applied melatonin.

## 2. Materials and Methods

### 2.1. Preparation of Plant Materials

Seeds of maize (*Z. mays*) were washed and soaked in distilled water for 16 h. Imbibed seeds were planted on a modified paper roll system [33] using Korean calligraphy paper (80 cm × 15 cm). Soaked maize seeds were arranged in a line with approximately 6-cm spacing between seeds on the paper 2 cm below the long edge. Then, the paper was rolled up and set vertically, in a plastic culture vessel (6.5 cm × 6.5 cm × 9.5 cm) filled with 100 mL of distilled water. The prepared paper rolls were placed in a plastic box (30 cm × 26 cm × 19 cm), which was wet with distilled water to keep inside humid and sealed with a piece of plastic wrap with several holes. The maize seedlings were incubated at 27 °C (under a 16 h-light/ 8-h dark) cycle in a growth chamber for five days. 

### 2.2. Application of Melatonin, Hormones, and Other Chemicals

The incubation medium (20 mL) containing melatonin or other substances in distilled water was prepared in a conical tube (50 mL). Five-day-old maize seedlings were placed in the tube carefully, such that roots were inserted in the medium, but leaves were not in direct contact with the medium; generally, each tube contained five maize seedlings. The prepared tubes were fixed in a rack. The rack was placed in a plastic box (30 cm × 26 cm × 19 cm), which was incubated at 27 °C for the planned test periods. The box was generally kept open, except some experiments with partially covered boxes to shield plants from the airflow inside the growth chamber. Melatonin, gibberellic acid (GA_3_), ABA, salicylic acid (SA) and benzo-18-crown-6 (18-CR) were purchased from Sigma-Aldrich (St. Louis, MO, USA).

### 2.3. Quantification of Melatonin

The leaves of the maize seedlings were harvested and weighed. Portions of leaves (6 cm) cut from the tip were macerated in 1 mL of absolute ethanol, and the crude extracts were incubated at −20 °C for at least 1 h to precipitate insoluble materials. Cell debris and precipitates were removed by centrifugation (13,000 rpm × 20 min) at 4 °C. The supernatant was vacuum-dried until the volume was reduced to 200 μL in a Speed-Vac system (Centrivap Concentrator, Labconco, Kansas City, MO, USA). The concentrated extract was diluted in 400 μL of distilled water. Melatonin in the extract was chemically modified to an oxidative fluorescent product, *N*-[(6-methoxy-4-oxo-1,4-dihydroquinolin-3-yl)methyl]acetamide as described previously [34,35]. Briefly, 10 μL of 250 mM H_2_O_2_ and 40 μL 2 M CaCO_3_ were added to the diluted solution (600 μL), and then the mixture was heated at 100 °C for 30 min. Products were partitioned with 600 μL ethyl acetate, and the organic phase collected. Partitioning was carried out for two additional rounds, and the organic phase collected was completely dried in a Speed-Vac system (Centrivap Concentrator, Labconco). The dried sample was dissolved in 10 μL acetonitrile and used for high-performance liquid chromatography (HPLC) analysis.

Modified melatonin was resolved on a RP-C18 HPLC column (Apollo C18, 5 μm, Alltech Associates, Deerfield, IL, USA), fitted to a pump system (Waters 600E, Waters, Milford, CT, USA), using an isocratic elution at a flow rate of 1.0 mL/min. The eluting solvent was prepared by mixing solution A (10% methanol containing 0.3% acetic acid) and B (90% methanol containing 0.3% acetic acid) at a ratio of 9:1. The elution profile was traced with a fluorescence monitor (Waters 470; excitation wavelength = 247 nm; emission wavelength = 392 nm). The melatonin peak was identified based on the comparison with a modified melatonin standard, and confirmed by the repeat analysis of the same analytical sample after the addition of the modified standard. The amount of melatonin was estimated by comparing the peak area with a standard curve that showed linear correlations (*R*^2^ = 0.9992) after chemical modification for effective fluorescence detection [34,35].

### 2.4. Observation of Stomata

The abaxial surface of maize leaves was magnified and images were captured with ITProTM software (Sometech, Seoul, Korea). To measure the stomatal pore area, stomata were examined at a magnification of 400× under a light microscope (Axiostar Plus, Zeiss, Oberkochen, Germany) connected to a digital camera (PowerShot G6, Canon, Tokyo, Japan). The stomatal pore area was analyzed with the Windows version of ImageJ software bundled with Java 8 (https://imagej.nih.gov/ij/), ImageJ is an open tool for scientific image analysis [36].

## 3. Results

**Figure 1:**
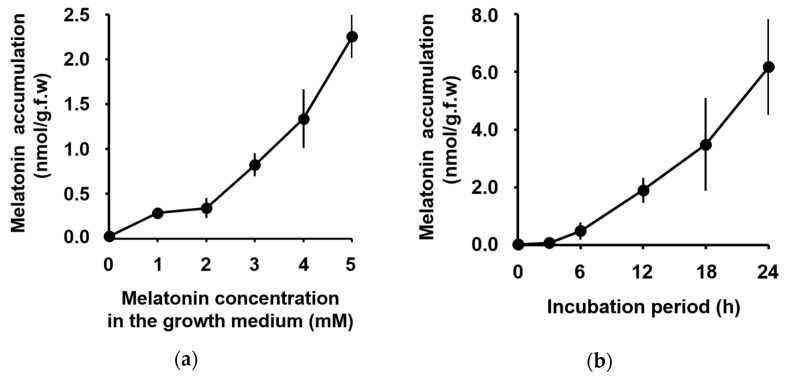
Foliar accumulation of melatonin applied to the roots of maize seedlings. (**a**) Correlation between melatonin accumulation in maize leaves and concentration of melatonin supplied in the growth medium. Melatonin accumulation was measured after 12 h from the melatonin application. (**b**) Time-dependent melatonin accumulation was measured after 4 mM melatonin was supplied. Data represent the mean ± standard deviation (SD) of six (**a**) and three (**b**) biological replicates. Each measurement was carried out with five seedlings.

The accumulation of exogenously applied melatonin was measured by the HPLC system fitted with an RP-C18 column, as described by Hamase et al. [34,35]. The level of melatonin in maize leaves increased proportionally to the concentration of melatonin supplied in the incubation medium (Figure 1a). Melatonin was first detected in the maize leaves 3 h after application, and then increased continuously up to 24 h (Figure 1b).

**Figure 2:**
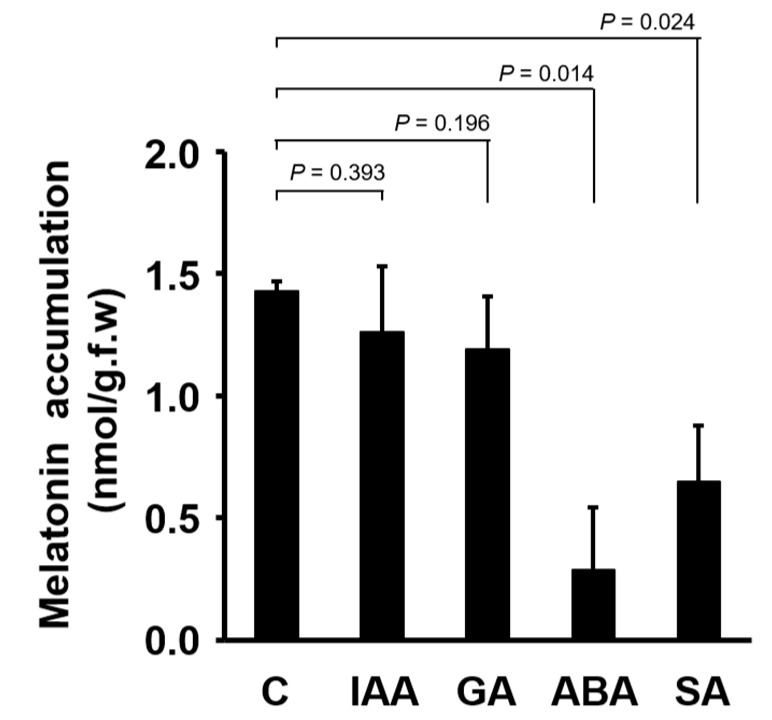
Influence of plant hormones on melatonin accumulation in maize leaves. The concentration of each hormone was 10 μM and that of melatonin was 4 mM. Melatonin accumulation was measured after 12 h. Data represent the mean ± SD of three biological replicates. Each measurement was carried out with five seedlings. Statistical significance was examined by Student’s *t*-test. Abbreviations: C, control; IAA, indole-3-acetic acid; GA, GA_3_; ABA, abscisic acid; SA, salicylic acid.

To determine whether plant hormones regulate the accumulation of melatonin in foliar tissues, we added indole-3-acetic acid (IAA), GA_3_, ABA and SA to the incubation medium. Indole-3-acetic acid and GA_3_ did not show any effect on the accumulation of melatonin in maize leaves (Figure 2). However, ABA and SA significantly decreased the amount of melatonin in leaves compared with the control (Figure 2).

**Figure 3:**
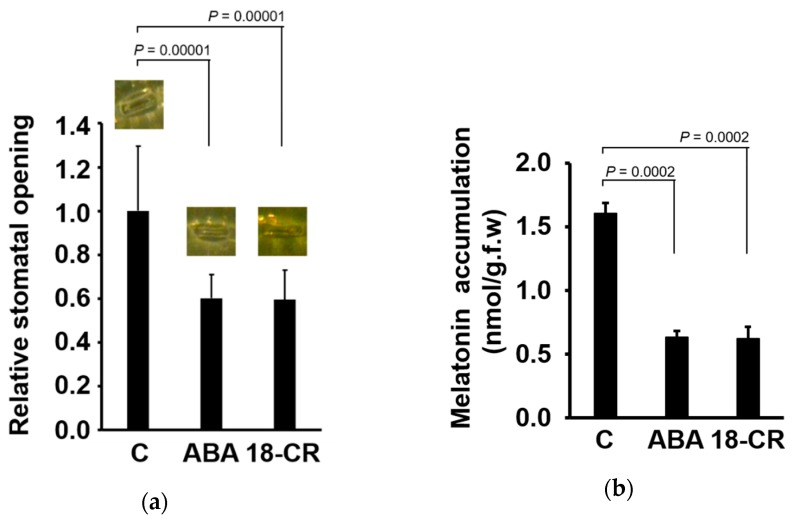
Effect of ABA and benzo-18-crown-6 (18-CR) on stomatal closure and melatonin accumulation. (**a**) The area of stomatal aperture was measured using a digital image equipment and analyzed with ImageJ. (**b**) Effect of ABA and 18-CR on melatonin accumulation in maize leaves. The concentrations of ABA and 18-CR were 10 μM and 0.1 mM, respectively. Plants were incubated in the presence of 4 mM melatonin for 12 h. Data represent the mean ± SD of at least 18 samples. Statistical significance was determined using Student’s *t*-test.

As both ABA and SA induce the closure of stomata [37], we investigated the relationship between stomatal closure and melatonin accumulation, following melatonin application, using ABA and 18-CR. Benzo-18-crown-6 is a chemical used to induce stomatal closure [38,39]. Both ABA and 18-CR reduced the stomatal pore area significantly (Figure 3a). In the same sample, subsequent analysis of melatonin accumulation revealed significant decreased of melatonin accumulation (Figure 3b).

**Figure 4:**
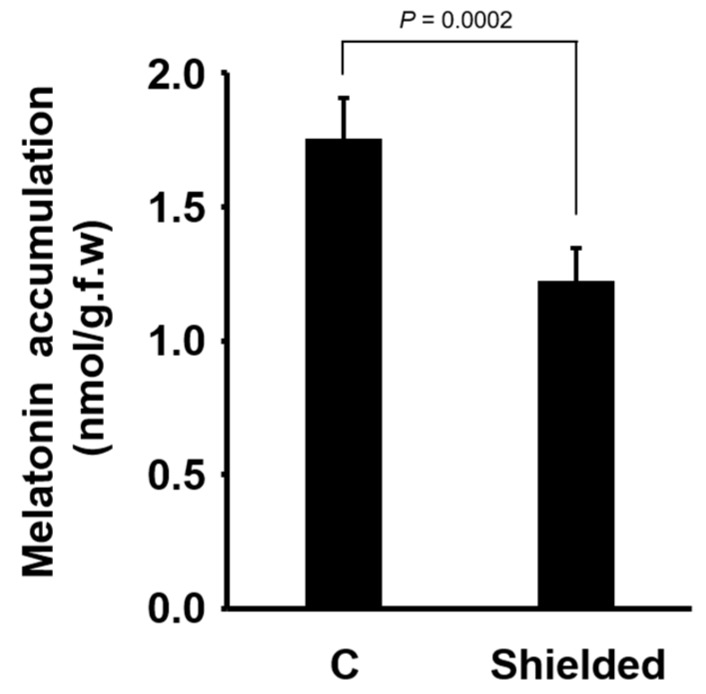
Melatonin accumulation in the control and shielded plants 12 h after melatonin (4 mM) application to the roots. Plants were shielded to reduce airflow. Data represent the mean ± SD (*n* = 20) originated from repeated experiments. Each test was carried out with two biological replicates containing five seedlings. Statistical significance was determined using Student’s *t*-test.

As stomatal resistance is the main factor affecting transpiration [40], we investigated the influence of transpiration on the accumulation of exogenously applied melatonin. Transpiration is higher under faster airflow and lower in stagnant air. To determine the effect of decreased airflow, we shielded the plants by loosely covering the incubation box. The accumulation of melatonin in shielded plants, protected from airflow in the growth chamber, was significantly lower than that in control plants (Figure 4).

## 4. Discussion

When melatonin was supplied to the roots of maize seedlings through the incubation medium, melatonin was detected in maize leaves. This is consistent with a previous report [22]. Although it is possible that exogenously applied melatonin promoted melatonin de novo biosynthesis in plants by increasing the expression of melatonin biosynthetic genes [30], the level of melatonin in leaves of maize seedlings supplied with exogenous melatonin was much higher than the endogenous levels in control plants. This suggests that the high level of melatonin in maize leaves was not solely because of increased melatonin biosynthesis due to exogenously applied melatonin.

An increase in the foliar accumulation of exogenously applied melatonin was proportional to the concentration of melatonin included in the incubation medium (Figure 1a). If any carrier or other apparatus that can be saturated at higher concentration of melatonin mediated the uptake of melatonin from the incubation medium, a saturated accumulation curve would be obtained. However, in our assay system, foliar accumulation of melatonin increased linearly (Figure 1b) and was not saturated. We did not observe any clue on a saturable melatonin uptake system. Additionally, the level of melatonin continuously increased over a 24-h period after the first peak at 3 h. An adaptable (or desensitizable) transport mechanism was not observed in the test period.

Phytohormones regulate diverse biological functions in plants either independently or together. Here, we determined the effects of several plant hormones on the accumulation of exogenously applied melatonin. While IAA and GA_3_ did not affect melatonin accumulation, ABA and SA significantly reduced the accumulation of melatonin in maize leaves compared with the control (Figure 2). 

Abscisic acid and SA have diverse functions. Although each hormone has its own action mechanisms, we noticed that both ABA and SA are commonly involved in the negative regulation of stomatal opening [37]. To determine whether the effect of ABA on the reduction in melatonin accumulation was due to stomatal closure, we tested the effect of another chemical, 18-CR, which directly closes the stomata [38,39]. Treatment with 18-CR induced stomatal closure and simultaneously decreased melatonin accumulation in the leaves (Figure 3). Therefore, the reduction in melatonin accumulation by ABA was explained by stomatal closure. The effect of SA on the decrease of melatonin accumulation could be explained also by stomatal closure. However, we cannot exclude the possibility that SA influences melatonin level in other ways, because melatonin regulates SA biosynthesis [41] and shows diverse interactions with SA [42].

As stomatal closure greatly influences transpiration, we tested whether a factor reducing transpiration would affect melatonin accumulation. Transpiration is high under streaming air and low in stagnant air. In the growth chamber used in this study, plants were exposed to constant airflow. We shielded the plants from the airflow by partially covering the incubation box with a plastic cap. Melatonin accumulation in shielded plants was significantly reduced, indicating that the transpirational flow is a determining factor for the foliar accumulation of exogenously applied melatonin (Figure 4). The influence of ethylene in a closed box, where ethylene produced from plants could be trapped, could be ruled out, because the box was not tightly closed, thus maintaining the outward diffusion of ethylene.

Recently, Erland et al. [32] used melatonin-conjugated quantum dots (QD-MEL) and showed that melatonin is absorbed through the epidermis and concentrated in endodermis and pericycle in *H. perforatum*. The authors also observed the upward movement of melatonin [32], consistent with our results in this study. However, the authors did not observe any sign of passive movement of melatonin with water through vascular tissues when roots were exposed to 4 pmol/L QD-MEL [32], which is much lower than the level of melatonin used in this study (1–5 mM). Increased melatonin exudation through the xylem of the watermelon (*Citrullus lanatus*) plants has been reported by another group after the application of up to 50 μM melatonin to the rhizosphere [11], indicating that melatonin absorbed by the roots moves upward through the xylem, following transpirational flow, when the concentration of the applied melatonin is sufficiently high. Both high and low affinity transport systems for low and high concentrations, respectively, of a regulatory molecule are known in auxin transport [43]. Two types of *N*-1-naphthylphthalamic acid (NPA)-binding sites with different K_d_ values are reported. Therefore, depending on the level of melatonin in plant tissues, different transport systems may be operative.

Furthermore, Li et al. [11] observed an increase in melatonin accumulation in roots of watermelon plants, when melatonin was sprayed on leaves. This phenomenon is difficult to explain with transpirational flow. Melatonin may move in plants through diverse systemic routes, depending on physiological conditions.

## 5. Conclusions

Melatonin applied exogenously to the root system of maize seedlings was absorbed and accumulated in the leaves. Melatonin accumulation was proportional to the applied concentration up to 5 mM, without saturation. Time-course analysis of the accumulated melatonin content did not show an adaptable (or desensitizable) uptake system over the 24-h period. Melatonin accumulation was reduced significantly by plant hormones ABA and SA, which commonly induce stomatal closure. ABA and 18-CR (a stomata-closing agent) induced stomatal closure and melatonin accumulation. Shielding the plants from continuous airflow in the growth chamber reduced melatonin accumulation in the leaves. Thus, stomatal closure mediated by ABA reduced transpiration, resulting in reduced foliar accumulation of exogenously applied melatonin. We conclude that melatonin applied exogenously to the root system was absorbed and transported to the leaves following the transpirational flow.
